# Personality, psychological stress, and self-reported influenza symptomatology

**DOI:** 10.1186/1471-2458-7-339

**Published:** 2007-11-23

**Authors:** Kim GE Smolderen, Ad JJM Vingerhoets, Marcel A Croon, Johan Denollet

**Affiliations:** 1 CoRPS-Centre of Research on Psychology in Somatic diseases, Tilburg University, Tilburg, The Netherlands; 2 Statistics and methodology Department, Tilburg University, Tilburg, The Netherlands

## Abstract

**Background:**

Psychological stress and negative mood have been related to increased vulnerability to influenza-like illness (ILI). This prospective study re-evaluated the predictive value of perceived stress for self-reported ILI. We additionally explored the role of the negative affectivity and social inhibition traits.

**Methods:**

In this study, 5,404 respondents from the general population were assessed in terms of perceived stress, personality, and control variables (vaccination, vitamin use, exercise, etc.). ILI were registered weekly using self-report measures during a follow-up period of four weeks.

**Results:**

Multivariable logistic regression analysis on ILI was performed to test the predictive power of stress and personality. In this model, negative affectivity (OR = 1.05, p = 0.009), social inhibition (OR = 0.97, p = 0.011), and perceived stress (OR = 1.03, p = 0.048) predicted ILI reporting. Having a history of asthma (OR = 2.33, p = < 0.0001) was also associated with ILI reporting. Older age was associated with less self-reported ILI (OR = 0.98, P = 0.017).

**Conclusion:**

Elderly and socially inhibited persons tend to report less ILI as compared to their younger and less socially inhibited counterparts. In contrast, asthma, trait negative affectivity, and perceived stress were associated with higher self-report of ILI. Our results demonstrate the importance of including trait markers in future studies examining the relation between stress and self-report symptom measures.

## Background

Together with the emergence of the field of psychoneuroimmunology, a growing interest can be observed in the role of psychosocial variables in the development of infectious disease, including the common cold and influenza-like illness [1]. From an etiological point of view, it is clear that whereas the pathogen should be considered as a necessary but not sufficient (e.g., dependent on its virulence) factor, psychosocial factors are neither necessary nor sufficient. The latter factors are rather hypothesized to affect the vulnerability of the host, and in such way contributing to increased risk of infectious disease. Psychological stress, often conceptualized as the exposure to major stressful life events, daily hassles, and negative mood has been associated with increased vulnerability to infectious illness [2-6]. For example, Graham *et al.*[6] found that life events and daily hassles were predictive for experiencing significantly more influenza-like symptoms (ILI). Cohen *et al.*[4,7,8] have also made some great efforts to unravel the precise nature of the relation between psychological stress, i.e., perceived stress, and susceptibility to upper respiratory illness. In his experimental inoculation trials, he repeatedly demonstrated that psychological stress is associated with increased risk for developing respiratory illness for persons intentionally exposed to a common cold virus, that the longer the duration of the stressor the greater the risk, and that the link between stress and susceptibility may be mediated by stress-induced disruption of the regulation of proinflammatory cytokines [4,7,8].

Other psychological predictors of interest are social support and social network diversity [3,9]. Multiple ties to friends, family, work, and community seem to be rather advantageous in terms of physical health [10,11]. However, having a diverse social network may not always be a positive factor. For example, Hamrick *et al.*[9] revealed that stress and diversity of social contacts interacted; among those who experienced more stressful life events, diversity was associated with more ILI. Cobb *et al.*[3] also failed to find support for a protective effect of social support in the stress – health relationship. This makes sense for infectious disease, since exposure to the pathogenic agents is more likely for individuals with a larger social network than for socially inhibited people.

In addition to broaden the focus on psychological predictors in the susceptibility to ILI, attention for a possible role of more basic personality styles seems to be interesting and promising in examining the relation between stress and infectious disease. It is important to document on determinants of, for example psychological distress and network diversity. Individual differences in personality, which result partly from genetic influences, significantly impact on the way in which humans structure the world around them [12]. These differences make individuals more or less likely to experience stressful events and to have poor quality interpersonal relationships, which in turn 'feed back' on the individual, influencing their risk for subsequent illness [13]. It is also crucial to focus on broader personality traits, such as negative affectivity (NA), because self-report measures of stress and health both contain a NA component [14]. Therefore, it seems advisable to include an established trait NA marker in health research, so that its influence can be identified and isolated. To date, there are only a few studies that have addressed the role of personality in studying vulnerability to infectious illness. For example, introversion has been identified as a predictor for developing significantly worse symptoms and infections after rhinoviruses exposure [15,16]. In other studies, NA was found to be associated with subsequent somatic complaints reporting [15,17-19].

Unfortunately, conceptual and methodological inconsistencies make it difficult to draw unambiguous conclusions about the role of personality traits in predicting susceptibility to ILI. In particular, the lack of naturalistic studies, reliable measures of personality traits, valid indicators of ILI, and the failure to take into account potentially relevant confounding variables, such as vitamin use and flu vaccination, are common shortcomings. Furthermore, the possible contributing role of other stable personality traits than introversion and negative affectivity did not receive adequate attention. Given these shortcomings, the inclusion of social inhibition (SI), or the tendency to inhibit the expression of emotions and behaviours in social interaction in a naturalistic study, may be a valuable addition to the current research in this area [20]. SI may be a relevant predictor of ILI, because it also proved to moderate the role of trait NA in other health outcomes [21-23].

To summarize, in the current study, we re-evaluated the value of perceived stress as a predictor of self-reported ILI. In addition, we explored the role of two personality traits, NA and SI as predictors of ILI report, while controlling for relevant confounders, such as vitamin use, smoking, flu vaccination, asthma, having pets, and regular exercise.

## Method

### Participants

Volunteers were recruited via a comprehensive media campaign to participate in this Dutch-Flemish project, entitled "De Grote Griepmeting" ("The Great Influenza Study") in the period running from November 2004 to February 2005. Our study was part of this greater study and people who registered for "The Great Influenza Study" were also asked to fill out psychological questionnaires. For this study, we followed participants who registered in the first week (week 46/2004) of the study for four consecutive weeks. In the first week, there were 13,964 registrations. There were 4,302 registered people who did not agree to fill out the psychological questionnaires, and 3,878 participants who did not provide data at all time points. Furthermore, participants who reported ILI at enrolment were excluded (n = 380), because of the possible confounding effect of illness on stress reporting, leaving 5,404 participants in our study. We found that non-responders were younger (40.9 years vs. 45.4 years, p < 0.0001) and tended to be more female (55.8% women in non-responders vs. 51.1% in responders, p < 0.0001). The study was conducted conform to ethical principles of the Helsinki Declaration and according to Dutch legislation on privacy. Following this legislation, written informed consent is not compulsory for observational studies. The privacy regulation of the study was approved by the Dutch Data Protection Authority.

### Measures

The following measures were applied:

#### Dependent variables

*ILI *– were determined by means of questions about ILI such as fever, coughing, and muscular pain (Table 1) [24]. A respondent was identified as a case suffering from influenza-like illness on the basis of the ILI definition of scientists from the Netherlands Institute for Health Services Research (NIVEL) and the National Institute for Public Health and the Environment (RIVM) in the Netherlands [24]. ILI was defined as fever >38°C, that started suddenly, plus headache or muscle pain, plus at least one respiratory symptom (running nose/coughing/sore throat/chest pain).

**Table 1:** Questions asked about symptoms of ILI

No Symptoms?	How high was the fever?
Running Nose?	Cough?
Sore Throat?	Headache?
Muscle Pain?	Chest Pain?
Abdominal Pain?	Diarrhoea?
Nausea?	Cold Shivers?
Sudden Fever? (No/Yes/don't know)	Irritated Eyes?

Table adopted from Marquet et al., 2006 [24]

#### Independent variables

*Perceived Stress *– was assessed by the 10-item Perceived Stress Scale (PSS). The PSS assesses the degree to which situations in one's life are appraised as stressful in the past month. The questionnaire has a good reliability and validity [25].

*Personality *– was measured by the type-D Scale-14 (DS14), a short questionnaire of 14 items with good psychometricproperties. The questionnaire measures NA and SI [26].

#### Control variables

In addition to sex, age, and flu vaccination; information about asthma, pets, vitamin use, smoking, and exercise was obtained. Information about flu vaccination, asthma, pets, vitamin use, smoking was scored as yes or no. Exercise was scored using the categories less than one hour per week or more than one hour per week.

### Procedure

The PSS and DS-14 were administered at baseline. ILI were recorded weekly on the basis of self-reports. Participants having registered themselves on the Internet site of "The Great Influenza Survey", received a weekly reminder requesting them to report their ILI online.

### Data analysis

Baseline characteristics of the sample were analysed stratified by the presence of reported ILI by means of univariate student's t-tests (continuous variables) and chi-square tests (dichotomous variables). We applied multivariable logistic regression (enter model) with personality and perceived stress as predictors and reported ILI within a follow-up period of four weeks as the outcome measure. In the analysis, we controlled for the potentially confounding effects of age, sex, asthma, pets, vitamin use, smoking, flu vaccination, and exercise. The control variables were analysed as dichotomous variables, except for age. The psychological variables were analysed as continuous variables.

All p-values were two-tailed, and p-values less than 0.05 were considered statistically significant. All analyses were conducted by using SPSS for Windows, version 12.0.1.

## Results

The baseline sample had a mean age of 45.8 (SD = 15.9 years), 49.9% (n = 2,697) was male. The baseline characteristics of the sample stratified by the presence of reported ILI are summarized in Table 2 (univariate analyses). ILI symptoms were reported in 343 (6.3%) participants. Negative Affectivity, perceived stress, female sex, younger age, asthma, and having pets was significantly associated with ILI reporting (p-values ranging from 0.001 to <0.0001).

**Table 2:** Baseline characteristics of the sample stratified by self-reported ILI (univariate analyses) (n = 5,404)

	**No ILI 93.7% (n = 5,061)**	**ILI 6.3% (n = 343)**	**P-value**
**Female Sex, % (n)**	49.5% (2,675)	59.1% (3,194)	*<0.0001*
**Mean Age ± SD**	46.1 ± 15.8	41.8 ± 16.3	*<0.0001*
**Asthma, % (n)**	6.1% (330)	14.2% (767)	*<0.0001*
**Pets, % (n)**	49.9% (2,697)	58.0% (3,134)	*0.001*
**Vitamin Use, % (n)**	49.0% (2,648)	52.6% (2,843)	0.140
**Smoking, % (n)**	19.2% (1,038)	21.7% (1,173)	0.203
**Flu Vaccination, % (n)**	24.2% (1,308)	27.5% (1,486)	0.117
**Exercise, % (n)**	53.2% (2,875)	52.1% (2,815)	0.657
**Mean Negative Affectivity **± SD****	8.9 ± 5.9	10.6 ± 6.0	*<0.0001*
**Mean Social Inhibition **± SD****	10.2 ± 6.4	9.9 ± 6.3	0.453
**Mean Perceived Stress **± SD****	13.8 ± 6.1	15.6 ± 6.1	*<0.0001*

ILI = Influenza-like Illness; SD = Standard Deviation

The multivariable logistic regression on reported ILI identified significant independent effects of age (p = 0.017), asthma (p < 0.0001), NA (p = 0.009), SI (p = 0.011), and perceived stress (p = 0.048). Asthma, NA, and perceived stress were associated with an increased probability of ILI reporting. In contrast, older age and SI predicted a decreased risk of ILI (Table 3).

**Table 3:** Results of the multivariable logistic regression analyses predicting ILI (n = 5,404)

**Determinant**	**OR**	**95%CI**	**P-level**
Female Sex	1.30	0.95–1.77	0.099
Age	0.98	0.98–1.00	*0.017*
Asthma	2.33	1.46–3.73	*<0.0001*
Pets	1.01	0.79–1.43	0.704
Vitamin Use	1.22	0.92–1.64	0.170
Smoking	0.90	0.62–1.29	0.544
Flu Vaccination	0.99	0.68–1.47	0.995
Exercise	0.92	0.69–1.23	0.574
Negative Affectivity	1.05*	1.01–1.08	*0.009*
Social Inhibition	0.97*	0.94–0.99	*0.011*
Perceived Stress	1.03*	1.00–1.07	*0.048*

*odds ratio (OR) per unit increase on the psychological variables is presented.

## Discussion

The aim of this study was to investigate the role of stress and personality, after having controlled for life style factors, in self-reported ILI. Older age and SI was associated with less self-reported ILI. Asthma, NA, and perceived stress were associated with more ILI reporting.

The emergence of advancing age as a protective factor of influenza-like illness might be surprising, as it appears that the elderly individuals are considered as an at-risk population for developing ILI and for influenza-related mortality [27]. However, in this study, self-report of ILI was used as an outcome measure and this may have yielded different results as one may expect to find when clinical outcome measures are used. A possible explanation for the emergence of age as a protective factor in this study, may be that the elderly actually underestimate their symptoms of illness in general, and of influenza-like illness in particular. Umachandran *et al.*[28] for example, found that in non-diabetic men with coronary artery disease, the perception of angina tends to deteriorate with advancing age, which was not solely attributable to alterations in autonomic function. Ladwig *et al.*[29] also found a slight decrease of somatic symptom reporting in higher age groups. The only study, to our knowledge, that examined age-related differences in self-report of ILI, was conducted by Thumin and Wims [30]. It was found that older respondents reported less contracting of influenza-like illness and perceived intestinal flu to be relatively less serious, as compared to younger participants. It might also be the case that our ILI definition was not sensitive to the experienced ILI symptoms in the elderly. Cox and Subbarao [31] state in their review that although several of the symptoms of ILI are common to all age-groups, the proportion of patients in whom these complaints are noted varies as a function of age.

Socially inhibited individuals reported less ILI than their more outgoing counterparts, which is in line with the previous findings of Cobb and Steptoe [3] and Hamrick *et al.*[9]. Socially inhibited persons may have smaller social networks, which decrease their risk of being infected by exposure via social contact. However, Cohen *et al.*[32,33] demonstrated in an experimental setting in which all participants were inoculated with a rhinovirus, that having more social ties and sociability were associated with less susceptibility to upper respiratory illness. This could probably occur because sociable people had better and closer relationships that might increase positive affect, promote positive health practices, and provide social support in stressful situations. Sociability was associated with positive affect in this paper and also in a later experimental study, Cohen *et al.*[8] showed that positive emotional style was associated with greater resistance to objectively verifiable upper respiratory infections.

On the other hand, in a naturalistic study of Hamrick *et al.*[9], having a more diversified social network was marginally associated with a greater incidence of verified upper respiratory infections. They also found that chronic stress, i.e. life events, and diversity of social contacts interacted; diversity was associated with more illnesses among those with more stressful life events and fewer illnesses among those with fewer stressful life events. Our study found that SI or the tendency to inhibit the expression of emotion and behaviour in social interaction and the tendency to be more socially isolated was associated with less symptom report. Exposure to a cold or influenza virus is probably more likely for individuals with a larger social network than for socially inhibited people. This finding is in accordance with the finding of Hamrick *et al.*[9]. Unfortunately, we were not in the possibility to test the associated interaction effect because we had no measure of chronic stress conceptualised as life events. It would be interesting to find out in future studies whether chronic stress and SI also interact in their relation with ILI.

The finding of increased ILI self-report for high NA individuals is also not without precedence. Cohen *et al.*[34] found that trait NA was associated with more disease-specific health complaints among people with viral respiratory illnesses, although this link was primarily attributable to over-reporting of disease symptoms. In addition, Leventhal *et al.*[19] found a connection between trait NA and somatic complaints. Although both studies reported that state in contrast to trait negative moods are more consistent predictors of later somatic complaints, in both cases, the investigators recognize that they have used a less than optimal measure of trait affect. In the current study, we used the NA subscale of the DS14, a personality assessment instrument with psychometrically sound properties. The DS14 subscales NA and SI are stable over time and both traits proved to represent major domains of personality both in general and in cardiac populations [26].

Perceived stress also predicted ILI reporting while controlling for personality variables and relevant control variables. This result is in line with the vast literature on psychological stress and increased vulnerability to infectious illness [2-6].

Having a history of asthma was also associated with an increased probability of reporting ILI in our study. This finding is in line with literature [35]. Likewise, it is known that people who have asthma are considered a high risk group for complications in influenza and for whom influenza vaccination is highly recommended [36].

It is important to stress some important weaknesses of the present study that may limit the generalizability of our results. First, our outcome measure was self-report. We cannot rule out the possibility that the relationships discovered may reflect merely differences in reporting behaviours rather than in actual symptomatology. Although the correspondence between self-reported ILI and objective measure of ILI has been questioned [37], several findings indicated a high level of agreement between self-report and objective assessment of illness [38,39]. Second, in spite of the large number of participants, due to self-selection and high drop-out rates, there can be no doubt that the sample is not an adequate representation of the population. We can only guess what the effects are of this for the researcher uncontrollable drop-out rates. Unfortunately, these drop-out rates seem to be inherent to internet based surveys [24,40]. Therefore, in future research, adequate attention should be paid to these aspects. In particular, another method of data sampling would be preferred, i.e. not via internet but with the classical paper-and-pencil method. Drawbacks of the latter method are a smaller sample that can be reached and possible reliability problems with self-report. Laboratory studies can overcome the reliability problems of self-report but are less naturalistic. Finally, the effects that were found are actually small but in line with previous findings [35].

## Conclusion

To summarize, the present study demonstrated that personality factors and psychological stress significantly predicted future self-reported ILI. Socially inhibited persons reported less ILI as compared to their less socially inhibited counterparts. Advancing age was also associated with less ILI symptom reporting. In contrast, asthma, trait NA, and perceived stress predicted higher self-report of ILI. Future research needs to focus on the precise nature of the relation between psychological factors and flu-like symptom reporting. For example, do psychological factors predict better in certain age groups? The present study emphasizes the relevance of including personality variables in future research on the relationship between psychological variables and ILI reporting.

## Competing interests

The author(s) declare that they have no competing interests.

## Authors' contributions

Four authors have contributed to the manuscript; KGES performed the statistical analyses of the data and drafted the manuscript, AJJMV and JD helped to draft the manuscript and have revised the manuscript critically for important intellectual content, and MC assisted with the statistical analysis of the data. All authors approved the final version of the manuscript.

## Pre-publication history

The pre-publication history for this paper can be accessed here:


